# Three-outcome designs for external pilot trials with progression criteria

**DOI:** 10.1186/s12874-024-02351-x

**Published:** 2024-10-02

**Authors:** Duncan T. Wilson, Eleanor Hudson, Sarah Brown

**Affiliations:** Clinical Trials Research Unit, Leeds Institute of Clinical Trials Research, University of Leeds, Leeds, LS2 9JT UK

**Keywords:** Pilot trial, Progression criteria, Sample size

## Abstract

**Background:**

Whether or not to progress from a pilot study to a definitive trial is often guided by pre-specified quantitative progression criteria with three possible outcomes. Although the choice of these progression criteria will help to determine the statistical properties of the pilot trial, there is a lack of research examining how they, or the pilot sample size, should be determined.

**Methods:**

We review three-outcome trial designs originally proposed in the phase II oncology setting and extend these to the case of external pilots, proposing a unified framework based on univariate hypothesis tests and the control of frequentist error rates. We apply this framework to an example and compare against a simple two-outcome alternative.

**Results:**

We find that three-outcome designs can be used in the pilot setting, although they are not generally more efficient than simpler two-outcome alternatives. We show that three-outcome designs can help allow for other sources of information or other stakeholders to feed into progression decisions in the event of a borderline result, but this will come at the cost of a larger pilot sample size than the two-outcome case. We also show that three-outcome designs can be used to allow adjustments to be made to the intervention or trial design before commencing the definitive trial, providing the effect of the adjustment can be accurately predicted at the pilot design stage. An R package, tout, is provided to optimise progression criteria and pilot sample size.

**Conclusions:**

The proposed three-outcome framework provides a way to optimise pilot trial progression criteria and sample size in a way that leads to desired operating characteristics. It can be applied whether or not an adjustment following the pilot trial is anticipated, but will generally lead to larger sample size requirements than simpler two-outcome alternatives.

**Supplementary Information:**

The online version contains supplementary material available at 10.1186/s12874-024-02351-x.

## Introduction

When there is some uncertainty about the feasibility of a planned randomised clinical trial (RCT), an external pilot trial can be conducted in advance [1, 2]. External pilots take the form of a smaller version of the main trial [3], and can be used to estimate various parameters of interest when deciding if (and how) to progress to the main study. Investing in a pilot trial can identify potential issues at an early stage, making a successful main trial more likely and reducing overall research waste [4].

Progression decisions are often guided by so-called *progression criteria* [5]. A single two-outcome progression criterion specifies a decision rule which maps the pilot data to a *stop* or *go* outcome. Specifically, the pilot data are used to calculate a statistic, typically an estimate of a parameter of interest, and this statistic is compared against a threshold value. If the statistic exceeds the threshold, the suggested decision is to *go* forward to the main trial; otherwise, to *stop* on the grounds of infeasibility. When progression criteria are specified for several parameters, these can be combined by proceeding to the main trial only if all of the estimates exceed their respective thresholds [6] or through more complex decision rules [7]. It has been recommended that, in addition to being reported in the pilot study manuscript, progression criteria are pre-specified at the protocol stage in agreement with the study funder [8, 9].

Progression criteria are often based on a three-outcome ‘traffic light’ system [10]. These criteria stipulate two threshold values for a given parameter of interest. If the estimate falls below the lower of these, the decision is to stop (red); if the estimate falls above the higher threshold, the decision is to proceed immediately to the main trial (green); and if the estimate falls between the two thresholds, an intermediate decision is reached (amber). The specific purpose and interpretation of this intermediate decision can vary, and will depend on the motivation for using the three-outcome system. Three such motivations can be found in the methodological literature.

The CONSORT 2010 statement argues that basing strict stop/go decisions on a single threshold may lead to an unacceptably high chance of making the wrong decision as a result of sampling variability [5]. Allowing for an intermediate result between *stop* and *go* may reduce the probability of incorrect decisions. A second motivation stems from the fact that many aspects (quantitative and qualitative) being studied in a pilot trial are potentially relevant to the progression decision, and that this decision will be jointly made by several stakeholders (such as the trial team, the trial steering committee, the funder, and patients). A three-outcome system will allow immediate *stop* or *go* decisions to be made if the evidence is sufficiently strong with respect to a handful of key parameters, whilst allowing, in the event of a borderline result, the decision to be informed by other data based on the differing perspectives of all decision makers. Sargent et al. argued this better represents what happens in practice even when a two-outcome process is nominally being followed. In that case, although a borderline result will technically dictate a firm *stop/go* decision, this may be overridden in light of other information [11].

A final reason for an intermediate outcome is to provide the flexibility needed to make some adjustment to the intervention or trial design in an attempt to improve the parameter in question and ensure the feasibility of the main trial. For example, after observing a mediocre follow-up rate in a pilot trial, the trial designers might consider moving from a postal follow-up strategy to one based on contacting the participants over the phone. A three-outcome approach could facilitate this by prescribing an ‘adjustment’ decision to the intermediate outcome, whilst still allowing for immediate stopping or progression when obtaining ‘stop’ or ‘go’ outcomes.

Despite the prevalence of quantitative three-outcome progression criteria [12], there is little statistical guidance to help researchers decide how to specify them. The related question of determining the pilot trial sample size is also undeveloped, with work in this area typically focusing on pilot trials where the primary objective is to estimate the primary outcome variance to inform the main trial sample size calculation. These methods are nevertheless used when this is not the main purpose of the pilot, often in the form of simple ‘rules-of-thumb’ [13–15]. Three-outcome designs have, however, been proposed in the setting of phase II trials of cancer treatments [16]. Some of these designs were motivated by the same factors given above, and so may provide a useful framework for the design and analysis of pilot trials with three-outcome progression criteria.

In this paper we consider if, and how, three-outcome phase II designs can be used to determine optimal progression criteria and sample size in pilot trials. We begin by introducing a simple example, before arguing that quantitative progression criteria are mathematically equivalent to hypothesis tests, and are best viewed as such. We then review relevant three-outcome phase II trial designs and extend these to the pilot trial setting. Finally, we examine the statistical properties of these pilot trial designs and consider whether or not they can help achieve any of the three motivating goals before concluding with a discussion.

## An example

Throughout this article we will refer to a simple example of a pilot trial assessing the probability that a participant in the intervention arm of the main trial will adhere to their prescribed treatment. Specifically, we consider adherence to be measured as a binary outcome and denote the probability of adherence by $\rho$. Given *n* patients in the pilot trial’s intervention arm, we then model the number of adherers using a binomial distribution with parameters $\rho$ and *n*. We denote the pilot estimate by $\hat{\rho }$.

We will consider both two- and three-outcome versions of progression criteria. In the two-outcome case, the progression decision is defined by a threshold value *x*, such that

1 $$\begin{aligned} \text {Decision} = \left\{ \begin{array}{ll} go & \text {if}\ \hat{\rho } \ge x \\ stop & \text {if}\ \hat{\rho } < x. \end{array}\right. \end{aligned}$$

In the three-outcome case, we allow for an additional intermediate result and require two thresholds, $x_0$ and $x_1$:

2 $$\begin{aligned} \text {Decision} = \left\{ \begin{array}{ll} go & \text {if}\ \hat{\rho } \ge x_1 \\ pause & \text {if}\ x_0< \hat{\rho }< x_1 \\ stop & \text {if}\ \hat{\rho } < x_0. \end{array}\right. \end{aligned}$$

The specific meaning of the intermediate *pause* result will vary depending on the purpose and context of the pilot trial.

## Progression criteria as hypothesis tests

In order to apply the two-outcome progression criteria of Eq. 1, we must choose the sample size *n* and the threshold *x*. One way to do so is though constructing a hypothesis test using the approach of A’Hern [17], as follows. First, we identify a parameter value $\rho _0$ such that if $\rho \le \rho _0$ we would like to limit the probability of incorrectly making a *go* decision (a type I error) to at most $\alpha ^*$. Similarly, we identify $\rho _1$ such that if $\rho \ge \rho _1$ we would like to limit the probability of incorrectly making a *stop* decision (a type II error) to at most $\beta ^*$. For example, we could choose adherence rates of $\rho _0 = 0.5$ and $\rho _1 = 0.7$ to represent poor and promising values respectively, and then use the standard choices of $\alpha ^* = 0.05, \beta ^* = 0.1$ for our nominal error rates. We then choose values of *n* and *x* which minimise *n* whilst satisfying the type I and II error rate constraints

3 $$\begin{aligned} \alpha & = \underset{\rho \le \rho _0}{\max} \Pr [ \hat{\rho }> x ~ | ~ \rho ] \nonumber \\ & = \Pr [ \hat{\rho } > x ~ | ~ \rho = \rho _0] \le \alpha ^* \end{aligned}$$

4 $$\begin{aligned} \beta & = \underset{\rho \ge \rho _1}{\max} \Pr [ \hat{\rho } \le x ~ | ~ \rho ] \nonumber \\ & = \Pr [ \hat{\rho } \le x ~ | ~ \rho = \rho _1] \le \beta ^*, \end{aligned}$$

where we have used the monotonicity of power as a function of $\rho$ to note that the type I and II error rates will be maximised when $\rho = \rho _0$ and $\rho = \rho _1$ respectively.

Alternatively, we can work backwards and take any given choice for *n* and *x* and calculate the resulting error rates for some hypotheses $\rho _0, \rho _1$. In particular, whenever a pilot trial progression criteria is specified in the form of Eq. 1, it is mathematically equivalent to a hypothesis test. For example, consider a pilot trial with $n = 15$ participants in the intervention arm and a *stop/go* progression criteria with threshold $x = 9/15$. If we suppose that the null and alternative hypotheses are $\rho _0 = 0.5, \rho _1 = 0.7$, this design will give type I and II error rates of $\alpha = 0.28$ and $\beta = 0.28$. If we instead constrain the error rates to, for example, $\alpha ^* = 0.05$ and $\beta ^* = 0.1$, the smallest possible sample size satisfying these constraints is $n = 53$ with a corresponding progression threshold is $x = 32/48$.

The equivalence of two-outcome progression criteria and hypothesis tests suggests the latter can provide a statistical framework for determining the former [6]. This will allow us to use hypotheses to express what parameter values would lead to errors of each type, and then subsequently to control the probability of these errors by choosing a sufficient sample size and associated progression threshold.

## Extending three-outcome phase II trial designs

Just as standard hypothesis testing can be used as a framework for two-outcome progression criteria, three-outcome extensions of it can be used for the three-outcome progression criteria of Eq. 2. We will consider two such extensions proposed for phase II trials by Sargent et al. [11] and by Storer [18].

**Fig. 1:**
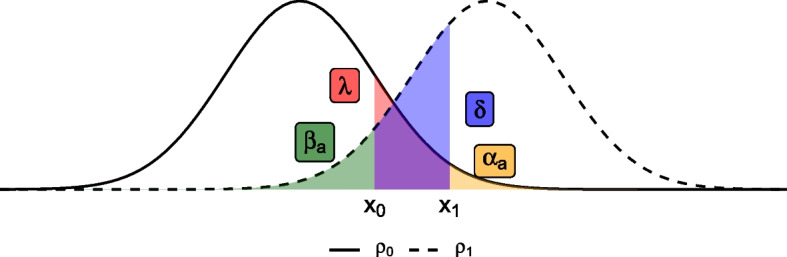
Graphical illustration of the operating characteristics for Sargent et al.’s three-outcome design [11]. The curves represents the sampling distribution of the estimate under the null hypothesis $\rho = \rho _0$ (solid line) and the alternative hypothesis $\rho = \rho _1$ (dashed line)

The design of Sargent et al. defines four operating characteristics relevant to the three-outcome setting. Firstly, a measure akin to the type I error rate, denoted $\alpha _a$, is defined as the probability under the null hypothesis $\rho = \rho _0$ that the parameter estimate will exceed the upper threshold $x_1$ and thereby lead to an incorrect *go* decision. Similarly, a type II error rate $\beta _a$ is given as the probability, under the alternative hypothesis $\rho = \rho _1$, of the parameter estimate falling below the lower threshold $x_0$ and leading to an incorrect *stop* decision. Two further operating characteristics relating to the intermediate outcome are then defined: the probability of obtaining a *pause* decision under the null hypothesis, denoted $\lambda$, and again under the alternative hypothesis, denoted $\delta$. These operating characteristics are summarised in Table 1 and illustrated in Fig. 1. The authors propose to set constraints on these four operating characteristics and choose $n, x_0,$ and $x_1$ to minimise *n* whilst satisfying these constraints. They argue that their designs will lead to a lower sample size requirement than standard two-outcome alternatives by reducing the probabilities of type I and II errors ($\alpha _a$ and $\beta _a$) through increasing the probabilities of *pause* decisions ($\lambda$ and $\delta$).

**Table 1:** Operating characteristics for Sargent et al. and Storer’s three-outcome designs [11, 18]

	Symbol	Equation	Description
Sargent	$\alpha _a$	$\Pr [ \hat{\rho } > x_1 | \rho = \rho _0]$	Probability of an immediate *go* decision under the null hypothesis
	$\beta _a$	$\Pr [ \hat{\rho } \le x_0 | \rho = \rho _1]$	Probability of an immediate *stop* decision under the alternative hypothesis
	$\lambda$	$\Pr [ x_0 < \hat{\rho } \le x_1 | \rho = \rho _0]$	Probability of a *pause* decision under the null hypothesis
	$\delta$	$\Pr [ x_0 < \hat{\rho } \le x_1 | \rho = \rho _1]$	Probability of a *pause* decision under the alternative hypothesis
Storer	$\alpha _b$	$\Pr [ \hat{\rho } > x_0 | \rho = \rho _0]$	Probability of not obtaining an immediate *stop* decision under the null hypothesis
	$\beta _b$	$\Pr [ \hat{\rho } \le x_1 | \rho = \rho _1]$	Probability of not obtaining an immediate *go* decision under the alternative hypothesis
	$\gamma _L$	$\Pr [ \hat{\rho } \le x_0 | \rho = \rho _m]$	Probability of an immediate *stop* decision when $\rho = \rho _m$
	$\gamma _U$	$\Pr [ x_1 < \hat{\rho } | \rho = \rho _m]$	Probability of an immediate *go* decision when $\rho = \rho _m$

**Fig. 2:**
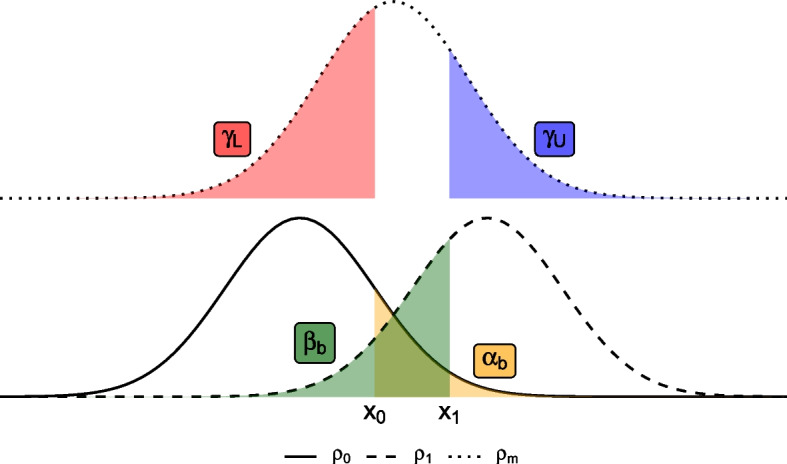
Graphical illustration of the operating characteristics for Storer’s three-outcome design [18]. The curves represents the sampling distribution of the estimate under the null hypothesis $\rho = \rho _0$ (solid line), the alternative hypothesis $\rho = \rho _1$ (dashed line), and a mid point $\rho = \rho _m = (\rho _1 + \rho _0)/2$ (dotted line)

An alternative three-outcome design proposed by Storer [18] takes the same basic approach, but with a different set of four operating characteristics. Here, the type I error rate $\alpha _b$ is taken to be the probability of exceeding the lower threshold, $x_0$, under the null; and similarly the type II error rate $\beta _b$ is now the probability of failing to exceed the upper threshold under the alternative. The remaining two operating characteristics are the probabilities of incorrectly obtaining a *stop* or a *go* decision when the true parameter is at some midpoint $\rho _m \in (\rho _0, \rho _1)$. These operating characteristics, denoted by $\gamma _L$ and $\gamma _U$ respectively, reflects the motivation of this design to *encourage* an intermediate outcome when the true parameter value is between the null and alternative. The operating characteristics are summarised in Table 1 and illustrated in Fig. 2, where we follow the author’s suggestion to set $\rho _m = (\rho _1 + \rho _0)/2$.

Considering the proposal of Sargent et al., we note that the measure $\alpha _a$ does not fully capture the probability of making a type I error since a decision to progress to the main trial can be arrived at in two ways: directly, by obtaining $\hat{\rho } > x_1$; or indirectly, by first obtaining a *pause* outcome $x_0< \hat{\rho } < x_1$ and then deciding to proceed. To capture these situations, we define the probabilities of making incorrect decisions following a *pause* outcome under the null and alternative hypotheses:

5 $$\begin{aligned} \eta _0 & = \Pr [\text {decide to}\ {go}\ |\ \rho = \rho _0, x_0 < \hat{\rho } \le x_1]\end{aligned}$$

6 $$\begin{aligned} \eta _1 & = \Pr [\text {decide to}\ stop\ |\ \rho = \rho _1, x_0 < \hat{\rho } \le x_1]. \end{aligned}$$

For example, $\eta _0$ is the probability of making a *go* decision following a *pause* outcome and when the true parameter value is $\rho _0$. The probability of making a *go* decision when $\rho = \rho _0$, then, is not $\alpha _a$ but

7 $$\begin{aligned} \alpha = \alpha _a + \eta _0 \lambda . \end{aligned}$$

Similarly, the type II error rate is

8 $$\begin{aligned} \beta = \beta _a + \eta _1 \delta . \end{aligned}$$

Previous authors have suggested these operating characteristics in the context of multi-armed screening trials [19, 20]. For simplicity we will assume that $\eta _0 = \eta _1 = \eta$; that is, the probability of eventually making the wrong decision following an intermediate result is the same when $\rho = \rho _0$ as when $\rho = \rho _1$. Under this reformulation an optimal three-outcome design can be found by first estimating the probability $\eta$, setting constraints on the type I and II error rates $\alpha , \beta$, and finally searching for the values of $n, x_0, x_1$ which minimise *n* whilst satisfying the constraints. Note that we no longer need to set constraints on the operating characteristics $\lambda$ and $\delta$, as these probabilities of obtaining an intermediate decision under the null and alternative hypothesis will be automatically limited by the constraints on $\alpha$ and $\beta$ (as defined in Eqs. 7 and 8) respectively.

A similar argument applies when considering Storer’s method, where we can replace the operating characteristics $\alpha _b, \beta _b$ with $\alpha$ and $\beta$. As the operating characteristics $\gamma _L, \gamma _U$ are designed to encourage an intermediate outcome under $\rho _m$, rather than limit it as in the method of Sargent et al., we keep these in our reformulation. Thus, an optimal three-outcome design under the reformulated Storer method can be found by estimating the probability $\eta$, setting constraints on $\alpha , \beta , \gamma _L$ and $\gamma _U$, and finally searching for the values of $n, x_0, x_1$ which minimise *n* whilst satisfying the constraints.

For simplicity, we will assume that the cost of an incorrect *stop* or *go* decision when $\rho = \rho _m$ are the same, and replace the two error rates $\gamma _U, \gamma _L$ by the single error rate

$$\begin{aligned} \gamma = \gamma _L + \gamma _U, \end{aligned}$$

the probability of making an incorrect conclusive decision of either type. Note that the reformulated method of Sargent et al. is a special case of this method when we set the trivial constraint $\gamma \le 1$, and so we have a single unified framework for designing and analysing three-outcome pilot studies which don’t allow for adjustments to the intervention or trial design prior to the definitive trial.

### Allowing for adjustments following a *pause* outcome

We now further generalise the three-outcome testing framework to allow for adjustments to be made following a *pause* outcome. Denote the effect of this adjustment by $\tau$, such that the parameter in the main trial will equal $\rho ' = \rho$ if no adjustment is made and $\rho ' = \rho + \tau$ if it is. We will assume that the adjustment effect is known up to an interval $\tau \in [\tau _{min}, \tau _{max}]$, and that $\tau _{min} \ge 0$. We then refine our definitions of the error rates $\alpha$ and $\beta$ as

- $\alpha$: the probability of proceeding to the main trial when $\rho ' \le \rho _0$
- $\beta$: the probability of not proceeding to the main trial when $\rho ' \ge \rho _1$;

Because we can make a *go* decision in two ways, $\alpha$ is now the maximum probability of proceeding either directly or following a *pause* outcome (in which case the adjustment is made) when this will lead to $\rho ' \le \rho _0$. As before, we assume there is a constant probability of mistakenly deciding to proceed following a *pause* outcome when in fact $\rho + \tau \le \rho _0$, denoted by $\eta$. That is,

$$\begin{aligned} \alpha = \max \left[ \underset{\rho \le \rho _0}{\max} \Pr (x_1< \hat{\rho }), \underset{\rho + \tau \le \rho _0}{\max} \eta \Pr (x_0< \hat{\rho } \le x_1) + \Pr (x_1 < \hat{\rho }) \right] . \end{aligned}$$

The first term is maximised at $\rho = \rho _0$. The second term can be written as

$$\begin{aligned} \eta \Pr (x_0< \hat{\rho } \le x_1) + \Pr (x_1 < \hat{\rho }) & = \eta \Pr (\hat{\rho } \le x_1) - \eta \Pr (\hat{\rho } \le x_0) + 1 - \Pr (\hat{\rho } \le x_1)\\ & = 1 + (\eta - 1)\Pr (\hat{\rho } \le x_1) - \eta \Pr (\hat{\rho } \le x_0), \end{aligned}$$

and so is maximised at $\rho = \rho _0 - \tau _{min}$, giving

9 $$\begin{aligned} \alpha & = \max \big [ \Pr (x_1< \hat{\rho } ~|~ \rho = \rho _0), ~\eta \Pr (x_0< \hat{\rho } \le x_1 ~|~ \rho = \rho _0 - \tau _{min}) \nonumber \\ & \quad + \Pr (x_1 < \hat{\rho } ~|~ \rho = \rho _0 - \tau _{min}) \big ]. \end{aligned}$$

An incorrect *stop* decision may again occur two ways - directly, or following a *pause* outcome. The error rate $\beta$ can therefore be written as

10 $$\begin{aligned} \beta = \max \left[ \underset{\rho > \rho _1}{\max} \Pr (\hat{\rho } \le x_0), \underset{\rho + \tau > \rho _1}{\max} \Pr (\hat{\rho } \le x_0) + \eta \Pr (x_0 < \hat{\rho } \le x_1) \right] . \end{aligned}$$

The first term is maximised at $\rho = \rho _1$, while the second term can be written as

$$\begin{aligned} \eta \Pr (\hat{\rho } \le x_1) + (1 - \eta ) \Pr (\hat{\rho } \le x_0), \end{aligned}$$

which is maximised at $\rho = \rho _1 - \tau _{max}$. Since $\tau _{max} > 0$, then $\Pr (\hat{\rho } \le x_0 | \rho = \rho _1) \le \Pr (\hat{\rho } \le x_0 ~|~ \rho = \rho _1 - \tau _{max})$ and so Eq. 10 can be simplified to

11 $$\begin{aligned} \beta = \Pr (\hat{\rho } \le x_0 ~|~ \rho = \rho _1 - \tau _{max}) + \eta \Pr (x_0 < \hat{\rho } \le x_1 ~|~ \rho = \rho _1 - \tau _{max}). \end{aligned}$$

Note that the general error rates of Eqs. 9 and 11 are valid in the special case where no adjustments are going to be made following a *pause* decision (i.e. when $\tau _{min} = \tau _{max} = 0$). As such, we have a general formulation of error rates in three-outcome designs regardless of the possibility of adjustment.

### Implementation

To determine appropriate values for $n, x_0$ and $x_1$ which will satisfy nominal error rate constraints $\alpha ^*, \beta ^*$ and $\gamma ^*$, we first consider *n* and $x_1$ to be fixed and such that $\Pr (\hat{\rho } > x_1 ~|~ \rho = \rho _0) \le \alpha ^*$. Then we set the second term in Eq. 9 equal to $\alpha ^*$ and rearrange to get

12 $$\begin{aligned} \Pr (\hat{\rho } \le x_0) = \frac{1}{\eta } + \frac{\eta - 1}{\eta }\Pr (\hat{\rho } \le x_1) - \frac{\alpha ^*}{\eta }. \end{aligned}$$

Using the inverse of $\hat{\rho }$’s distribution function, we can then find the value of $x_0$ which gives us $\alpha = \alpha ^*$ (or for the case of a binary outcome, the $x_0$ which maximises $\alpha$ whilst ensuring $\alpha \le \alpha ^*$).

To choose $x_1$ we continue to fix *n* and then use a numerical search to find the largest $x_1$ such that $x_0 \le x_1$ (with $x_0$ determined using Eq. 12) and $\beta \le \beta ^*$. Finally, we can choose *n* by finding the smallest value such that the third constraint $\gamma \le \gamma ^*$ is satisfied when the corresponding $x_1$ and $x_0$ are chosen using the above procedure.

The R package tout uses this approach to determine optimal three-outcome designs for the case of univariate progression criteria based on a binary or continuous endpoint in single arm, single stage trials. See the supplementary material and package documentation for full details and illustrations.

## Evaluation

As noted in the introduction, adding a third outcome to pilot trial progression criteria has been motivated on grounds of i) statistical efficiency; ii) the need to incorporate other information or stakeholders into progression decisions; and iii) the need to make modifications to the intervention or the trial design before commencing the main trial. In this section we examine to what extent the three-outcome design framework described in the previous section can be used to meet these goals.

### Statistical efficiency

**Fig. 3:**
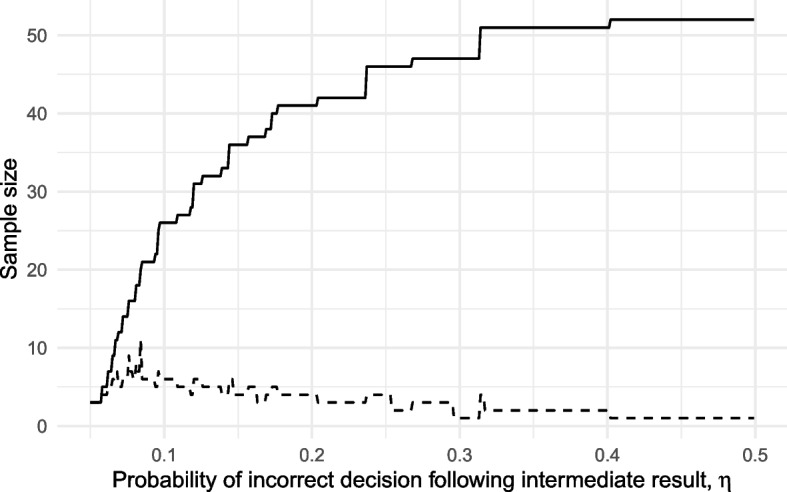
Minimum required sample size for a three-outcome design as a function of $\eta$ (solid line), along with the corresponding size of the intermediate zone $x_1 - x_0$ (dashed line)

Sargent et al. show that three-outcome designs based on the operating characteristics given in Table 1 will have a lower sample size than corresponding two-outcome designs whilst constraining the error rates $\alpha _a, \beta _a$ to the same levels, providing $\lambda$ and/or $\delta$ are allowed to be greater than 0. For example, take $\rho _0 = 0.5$ and $\rho _1 = 0.7$. A two-outcome design will require $n = 53$ to ensure $\alpha _a \le 0.05$ and $\beta _a \le 0.1$. In contrast, by allowing $\lambda \le 0.1$ and $\delta \le 0.1$ in a three-outcome design, we can obtain $\alpha _a \le 0.05$ and $\beta _a \le 0.1$ with only $n = 42$, suggesting three-outcome designs are indeed more efficient [11, 21]. However, this apparent advantage breaks down when using our reformulation. To illustrate this we found optimal sample sizes for this example problem over the range $0 \le \eta \le 0.5$ (where $\eta$ denotes the probability of making an incorrect progression decision following a *pause* outcome), using the constraints $\alpha \le 0.05, \beta \le 0.2, \gamma \le 1$. We have not considered $\eta > 0.5$ as this represents a decision-making ability worse than random, in which case the optimal design remains the usual two-outcome design. The optimal sample sizes are plotted in Fig. 3, where the discrete nature of sample size leads to step functions.

When $\eta = 0.5$, in which case we can only guess at the correct decision following a *pause* outcome, the optimal sample size is $n = 52$. Figure 3 shows that a low value of $\eta$ is required to achieve a meaningful reduction in sample size. For example, for a 20% reduction from the $n = 52$ two-outcome design down to $n = 41$, we would require $\eta = 0.2$. That is, we must be confident that following a *pause* outcome, but with a true $\rho = \rho _i (i = 0,1)$, we will make the correct progression decision with a probability of 0.8. In the context of our simple one-parameter example, the estimate $\hat{\rho }$ is a sufficient statistic for $\rho$ and so we cannot obtain any other information relevant to this particular judgement. This would lead to $\eta = 0.5$, in which case the optimal three-outcome design will reduce to a two-outcome design.

We may expect $\eta < 0.5$ if measures of another outcome in the trial, correlated with the outcome of interest, are going to inform the progression decision following a *pause* outcome. For example, patient adherence and retention may be correlated. If a *pause* outcome was observed when assessing adherence but retention was seen to be high, we might infer the true adherence rate to be larger than the estimate. The extent of this will depend, however, on the strength of the correlation, which may be hard to judge at the design stage.

**Fig. 4:**
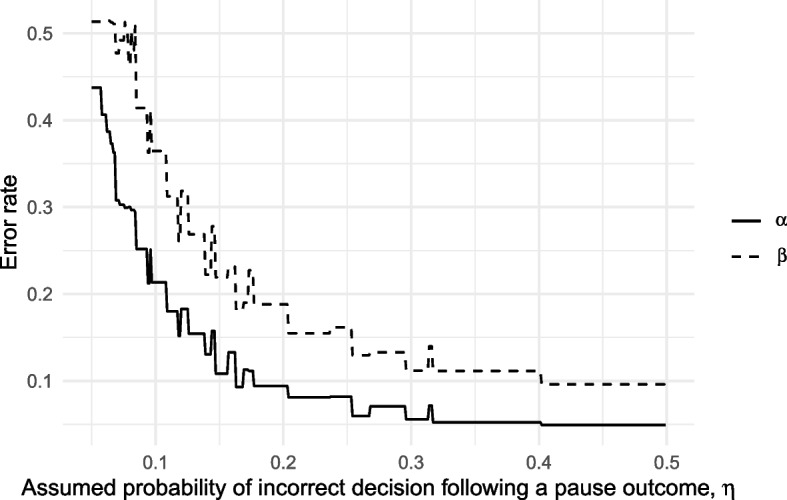
Type I (solid line) and II (dashed line) error rates of three-outcome designs, each locally optimal for an assumed $\eta$, when in fact $\eta = 0.5$

To explore the implications of incorrectly assuming $\eta < 0.5$, we took each of the optimal designs found over the range $0 \le \eta \le 0.5$ and calculated their type I and II error rates under a true value of $\eta = 0.5$. These error rates are plotted in Fig. 4. We find that error rates will be substantially inflated whenever $\eta$ was incorrectly assumed to be small enough to lead to a meaningful reduction in sample size. For example, if we incorrectly assume $\eta = 0.2$ when in fact $\eta = 0.5$ the ‘optimal’ design will lead to actual type I and II error rates of 0.094 and 0.188, rather than the nominal 0.05 and 0.1.

Given the challenges of estimating $\eta$ and the implications of doing it badly, we follow previous suggestions [19, 20] that a default assumption of $\eta = 0.5$ is appropriate. Under this conservative assumption, three-outcome progression criteria will not improve statistical efficiency in pilot trials beyond two-outcome alternatives.

### Incorporating other information

Although three-outcome progression criteria are not more efficient than their two-outcome alternatives, we may nevertheless wish to use them to allow other information to inform the progression decision rather then being ignored completely. For example, in addition to requiring sufficient adherence we may also want to see good recruitment and retention in the pilot trial. In the event of a *pause* outcome when assessing adherence, we could then decide to proceed to the main trial only if the estimated recruitment and retention rates are large enough. A *pause* outcome could also provide an opportunity for discussion amongst the various stakeholders (such as the trial team, steering committee, funder, and patients) to arrive at a collective decision on progression.

**Fig. 5:**
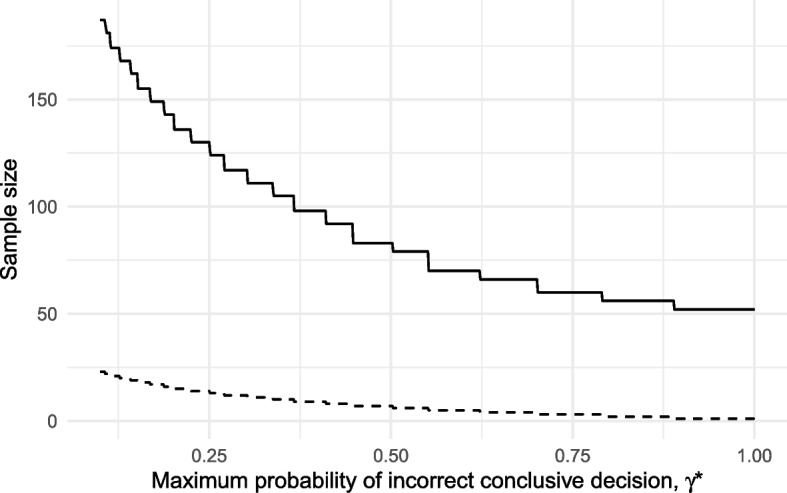
Minimum required sample size for a three-outcome design as a function of $\gamma ^*$ (solid line), along with the corresponding size of the intermediate zone $x_1 - x_0$ (dashed line)

To facilitate this we can encourage the design to have an appropriate intermediate zone $| x_1 - x_0|$ by constraining the operating characteristic $\gamma = \gamma _L + \gamma _U$ defined in Table 1, thus limiting the chance of making a conclusive *stop* or *go* decision when $\rho = \rho _m$. With $\rho _0 = 0.5, \rho _1 = 0.7, \alpha \le 0.05$ and $\beta \le 0.1$ as before, we set $\rho _m = (\rho _1 - \rho _0)/2 = 0.6$ and found optimal designs for a range of $\gamma ^*$ while assuming throughout that $\eta = 0.5$. The sample sizes of these designs are plotted in Fig. 5.

When we set $\gamma ^* = 1$, no intermediate zone is required and so the optimal design is the usual two-outcome design. As we decrease the nominal level on this constraint we permit an ever smaller probability of obtaining a conclusive *stop* or *go* outcome when $\rho = \rho _m$. This leads to an increasing width of intermediate zone $|x_1 - x_0|$, alongside an increasing sample size. The required increase in sample size beyond the two-outcome design can be substantial. For example, to ensure a maximum 40% chance of obtaining a conclusive result when $\rho = \rho _m$, we must increase the sample size from $n = 52$ to $n = 98$. Providing such increases in sample size are considered worthwhile, we conclude that three-outcome designs can be used in pilot trials to allow other information and stakeholders to feed into progression decisions.

### Allowing for adjustments

A final rationale for an intermediate outcome in pilot trials is to enable some modifications to be made prior to commencing the main trial. These could be adjustments to the trial design (e.g. to improve recruitment) or to the intervention itself (e.g. to improve adherence). The intermediate *pause* outcome now leads to the decision to either *stop* or to make these modifications and then *go* to the main trial. Recall that the effect of such an adjustment is denoted by $\tau$.

#### Known adjustment effect

**Fig. 6:**
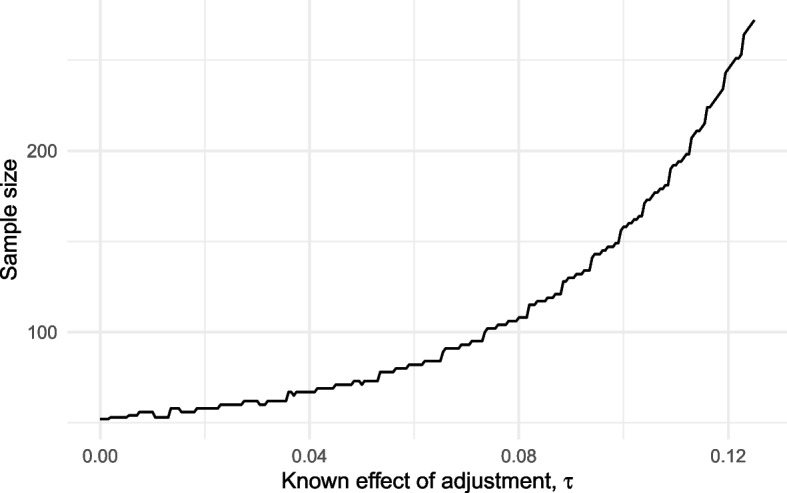
Minimum required sample size for a three outcome design as a function of the known adjustment effect $\tau$

Assume that the effect of adjustment $\tau$ is known a priori. Considering the same problem as before ($\rho _0 = 0.5, \rho _1 = 0.7, \alpha ^* = 0.05, \beta ^* = 0.1, \eta = 0.5$) we found optimal designs for a range of known adjustment effects spanning $\tau \in [0, 0.125]$. The required sample size of these designs is illustrated in Fig. 6.

When adjustments have no effect ($\tau = 0$), the optimal three-outcome design reduces to the usual *stop/go* two-outcome design with $n = 52$. As $\tau$ increases the required sample size increases with it exponentially. For example, for $\tau = 0.125$ we require $n = 275$. This can be explained by looking back at our error rate definitions in Eqs. 9 and 11, which show that $\alpha$ constrains the term $\Pr (x_1 < \hat{\rho } ~|~ \rho = \rho _0)$ and thus places a lower limit on $x_1$; meanwhile, $\beta$ constrains the term $\Pr (\hat{\rho } < x_0 ~|~ \rho = \rho _1 - \tau _{max})$ and thus forces $x_0$ to be lowered as $\tau _{max}$ increases, leading to a larger intermediate zone and correspondingly worse error rates.

#### Partially known adjustment effect

**Fig. 7:**
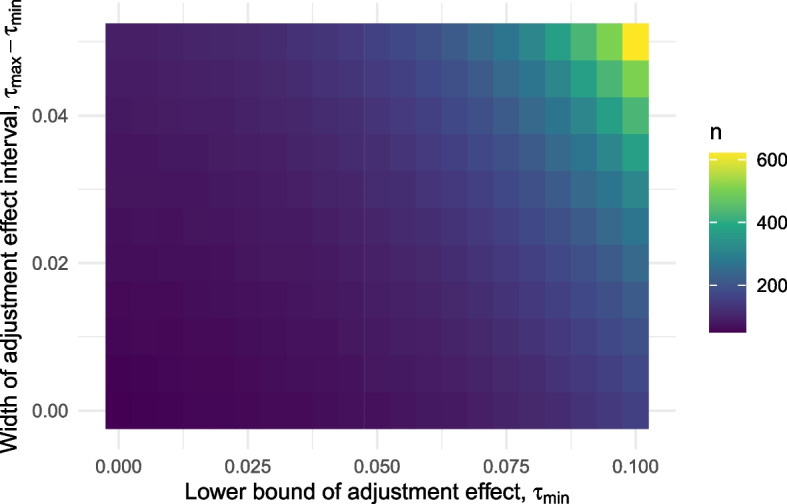
Minimum required sample size for a three outcome design as a function of the lower limit of the adjustment effect $\tau _{min}$ and the width of the effect interval, $\tau _{max} - \tau _{min}$

We now consider the case where the adjustment effect $\tau$ is known only up to an interval $\tau \in [\tau _{min}, \tau _{max}]$. We considered a range of values for $\tau _{min}$ from 0 up to 0.1 alongside a range of interval widths $\tau _{max} - \tau _{min}$ from 0 to 0.05. The resulting sample sizes are plotted in Fig. 7.

We see that increasing the width of the adjustment effect interval leads to an increased sample size, and the rate at which this happens increases with the lower interval limit $\tau _{min}$. For example, moving from $[\tau _{min}, \tau _{max}] = [0, 0]$ to [0, 0.05] leads to a change in sample size from $n = 52$ to $n = 93$; while moving from $[\tau _{min}, \tau _{max}] = [0.1, 0.1]$ to [0.1, 0.15] takes us from $n = 158$ to $n = 620$. Figure 7 suggests that the main driver of sample size is the upper limit $\tau _{max}$, but changing $\tau _{min}$ while keeping this fixed can still lead to considerable changes in *n*. From example, $[\tau _{min}, \tau _{max}] = [0.05, 0.05]$ (that is, a known $\tau = 0.05$) requires $n = 71$ while $[\tau _{min}, \tau _{max}] = [0.01, 0.05]$ requires $n = 87$.

#### The case $\eta < 0.5$

In the preceding subsections we have assumed $\eta = 0.5$. In the context of allowing adjustments we might argue that $\eta < 0.5$ is plausible because, although we may not be able to learn anything about $\rho$ beyond what is provided by $\hat{\rho }$, we might learn something about the adjustment effect $\tau$ during the pilot. If we can assume this will reduce $\eta$, this will lead to a corresponding reduction in sample size in a manner similar to the no-adjustment case discussed previously (see supplementary material for more details). We must bear in mind, though, that learning about $\tau$ can only take us so far. Even in the extreme case where we can learn $\tau$ exactly, the residual uncertainty about $\rho$ will place a lower limit on our ability to make the correct decision following a *pause* outcome.

We conclude that three-outcome progression criteria can be used to allow for adjustments, but note that this ability will come at the cost of an increased sample size. This increase can be especially large if the effect of adjustment may be substantial, or if there is considerable uncertainty about how large it is.

## Discussion

We have shown how the three-outcome progression criteria commonly used in pilot trials can be viewed as three-outcome hypothesis tests, and described how related clinical trial designs from the phase II setting can be used (with some reformulation) to optimise these criteria and the pilot sample size. This allowed for a formal comparison to be made between three- and two-outcome designs for pilot trials, with the latter as a special case of the former. We have shown that three-outcome designs do not improve efficiency in comparison to two-outcome alternatives, but that three-outcome designs can be used to allow for a more realistic decision-making process involving multiple sources of information and multiple stakeholders in the event of a borderline result. We have also shown that three-outcome progression criteria can facilitate making adjustments to the intervention or trial design following the pilot when the effect of such an adjustment is known in advance. We found that there is a price to pay for these benefits, with three-outcome pilot trials needing a (sometimes considerably) larger sample size than two-outcome alternatives to obtain the same operating characteristics. This suggests that the small sample sizes typically seen in pilot trials [22] may be inadequate for their goals.

We have quantified the impact of using three-outcome progression criteria through the resulting required sample size while keeping operating characteristics constrained. An alternative would be to fix the pilot sample size and examine the impact on operating characteristics. For example, taking $\rho _0 = 0.5$ and $\rho _1 = 0.7$ as before and fixing $n = 30$, a two-outcome design with threshold $x_0 = x_1 = 17$ will give us operating characteristics of $\alpha = 0.18, \beta = 0.08, \gamma = 1$. Moving to a three-outcome design will allow us to reduce $\gamma$, but only at the expense of an increased $\alpha$ and $\beta$. For example, the design with $x_0 = 15$ and $x_1 = 20$ gives $\alpha = 0.22, \beta = 0.21, \gamma = 0.35$. Although this type I error rate is larger than conventional constraints, previous authors have argued that these may be relaxed in the setting of phase II [23, 24] and pilot [25] trials.

In order to apply the proposed method in practice we need to specify null and alternative hypotheses for the parameter of interest and put constraints on three error rates. Specifying hypotheses for feasibility parameters like adherence rates may be challenging when compared to the more typical setting of assessing efficacy. Generally speaking there will be no default choice for the null of a feasibility parameter, as opposed to the default efficacy null of no difference to standard care. Moreover, the concept of Minimal Clinically Important Difference, which often guides the choice of target difference in efficacy, will not apply to feasibility. It may help to begin by determining the midpoint $\rho _m = (\rho _0 + \rho _1)/2$ which we would consider a borderline value and where we would ideally like to obtain a *pause* outcome, then determine the width of the interval $|\rho _1 - \rho _0|$, and finally set constraints on error rates $\alpha$ and $\beta$ based on the relative impact of these errors when defined with respect to $\rho _0$ and $\rho _1$. Having determined these values, one can find optimal designs for a range of constraints on the third operating characteristic $\gamma$ as shown in Fig. 5. Alternatively, the choice of $\rho _0$ and $\rho _1$ could be driven by the corresponding impact on any subsequent trial as measured though, for example, its power [7].

In addition to defining hypotheses, the proposed method also requires values for $\eta _0$ and $\eta _1$, the probabilities of making an incorrect progression decision *with respect to the parameter* $\rho$ following a *pause* outcome under the null and alternative hypothesis respectively. We have argued that a default of $\eta _0 = \eta _1 = 0.5$ may be justified as a conservative assumption. Alternatively, we may anticipate a bias towards progressing from the pilot to the definitive trial. This could be modelled by setting $\eta _0 > 0.5, \eta _1 < 0.5$ whilst constraining $\eta _0 + \eta _1 = 1$, although we found this to have little impact on the optimal design in our running example.

Our results have highlighted the difficulties of allowing adjustments to be made following a pilot trial when using pre-specified progression criteria, even when the effect of the adjustment is known (or partially known) in advance. This may be an unrealistic assumption, since a primary goal of many pilot trials is to identify *unforeseen* problems and solutions to these. In this context, pre-specifying an upper threshold $x_1$ can help identify cases which are feasible enough, without modification, to proceed to the main trial. In contrast, the lower threshold $x_0$ appears somewhat arbitrary and may force inappropriate decisions. For example, if $x_0$ is set too high, we may be led to a *stop* decision (i.e. $\hat{\rho } \le x_0$) despite believing, based on what was seen in the pilot, that a certain modification would lead to an adjusted adherence rate greater than $\rho _1$. When a new and unanticipated adjustment is proposed following the pilot trial, a second (and potentially internal) pilot may be needed to establish that it works as expected. Brown et al. suggested a similar strategy in the context of phase II drug trials, and it is in line with guidance on the development and evaluation of complex interventions [26] which emphasises the iterative nature of the process. Alternatively, if the cost of adjustment is low then a two-outcome design could be used where we assume the modification will always be made following a *go* outcome. Note that the power of this design will depend on the unknown effect of adjustment $\tau$, and a conservative assumption of $\tau = \tau _{max}$ would lead to large sample size requirements.

The underlying statistical framework considered in this paper is frequentist, focused on pre-specified decision rules chosen based on their long-run operating characteristics. An alternative is to design and analyse the pilot trial under a Bayesian framework [27–29]. This could improve efficiency by allowing external information or expert knowledge to be incorporated into decision-making, and would enable a more flexible approach to analysis which could formally account for anticipated effects of adjustments based on what was seen in the pilot. Willan and Thabane [30] do not consider the question of optimising pilot sample size, but show through an example how a Bayesian analysis of pilot data can help quantify uncertainty around feasibility parameters by producing posterior distributions which can be used to design the main trial. A Bayesian approach would also help address the aforementioned difficulties in specifying parameters (including the the probability of making correct decisions following a *pause* outcome, and the anticipated effect of adjustment) by allowing uncertainty regarding these to be expressed through prior distributions.

Our work has been motivated by external pilot trials assessing the feasibility of a subsequent study, but may be equally relevant to other settings such as phase II drug trials where the parameter of interest is a measure of efficacy. For example, the three-outcome framework could be used when making post trial adjustments to improve efficacy by changing eligibility criteria in an attempt to focus on a subgroup of patients. Although our findings should also broadly apply to internal pilot trials, care may be needed when the error rates of the final analysis may be affected by a formal internal pilot analysis of a correlated endpoint (for example, adherence). Finally, we expect our conclusions to carry over from the univariate setting considered here to the more general multivariate setting, where several progression criteria are applied simultaneously [6], although it has been shown that such multivariate tests can be counter-intuitively inefficient [7].

## Supplementary Information

Additional file 1.

## Data Availability

Supplementary material, including all R code used to generate the results in this manuscript and the associated R package tout, can be found at https://github.com/DTWilson/tout.
