# Supplementary material for: Three-outcome designs for external pilot trials with progression criteria
Source: BMC Med Res Methodol. 2024 Oct 2;24:226. doi: 10.1186/s12874-024-02351-x (PMC11446087; doi:10.1186/s12874-024-02351-x)
Supplement: Supplementary file 1 — Additional file 1. [file 12874_2024_2351_MOESM1_ESM.pdf]

Three-outcome designs for external pilot trials with progression criteria - supplementary material

Duncan T. Wilson

2024-09-26

Table of contents

Introduction 1
Statistical efficiency 2
Incorporating other information 6
Allowing for adjustments 8
Relaxing error rate constraints while fixing n 13
Illustrative figures 14

```
library(tout)
library(ggplot2)
```

```
Warning: package 'ggplot2' was built under R version 4.3.3
```

Introduction

This document provides supplementary material to support the manuscript "Three-outcome designs for external pilot trials with progression criteria" by Wilson *et al.* In particular, it will show how the tout package can be used to re-create all of the results and plots of the manuscript.

You can install the development version of tout from GitHub with:

```
# install.packages("devtools")
devtools::install_github("DTWilson/tout")
```

For all our evaluations we will use an example problem where the outcome is binary with probability parameter ρ and the null and alternative hypotheses are $\rho_0 = 0.5, \rho_1 = 0.7$ respectively.

Statistical efficiency

The key parameter to vary here is η, the probability of making an incorrect decision when we have a pause outcome under the null or alternative hypothesis. When we set $\eta = 0.5$ the optimal three-outcome design reduces to a two-outcome design (at least approximately):

```
tout_design(rho_0 = 0.5, rho_1 = 0.7, alpha_nom = 0.05, beta_nom = 0.1,
            gamma_nom = 1, eta_0 = 0.5)
```

```
Three-outcome design

Sample size: 52
Decision thresholds: 31 32

alpha = 0.04918532
beta = 0.0959314
gamma = 0.8897466

Hypotheses: 0.5 (null), 0.7 (alternative)
Modification effect range: 0 0
Error probability following an intermediate result: 0.5 0.5
```

This makes sense - we don't want to have any chance of a *pause* outcome because we can't make reliable decisions following it, and we haven't asked for a meaningful constraint on the OC γ. If we reduce η, we will see some efficiencies:

```
tout_design(rho_0 = 0.5, rho_1 = 0.7, alpha_nom = 0.05, beta_nom = 0.1,
            gamma_nom = 1, eta_0 = 0.2)
```

```
Three-outcome design

Sample size: 41
Decision thresholds: 23 27

alpha = 0.04590215
beta = 0.1000021
```

```
gamma = 0.5377359

Hypotheses: 0.5 (null), 0.7 (alternative)
Modification effect range: 0 0
Error probability following an intermediate result: 0.2 0.2
```

So, we can reduce the sample size from 52 to 41 if we can assume $\eta = 0.2$. This might be quite optimistic; suppose we use $\eta = 0.4$:

```
tout_design(rho_0 = 0.5, rho_1 = 0.7, alpha_nom = 0.05, beta_nom = 0.1,
            gamma_nom = 1, eta_0 = 0.4)
```

```
Three-outcome design

Sample size: 51
Decision thresholds: 30 32

alpha = 0.04681809
beta = 0.1008189
gamma = 0.7809068

Hypotheses: 0.5 (null), 0.7 (alternative)
Modification effect range: 0 0
Error probability following an intermediate result: 0.4 0.4
```

We find that this is barely enough to move away from a two outcome design. Plotting the optimal sample size for a range of η gives

```
# Create a set of etas to evaluate
df <- data.frame(eta = seq(0.0501, 0.5, 0.001))

get_results <- function(eta) {
  # For any given eta, find the optimal design and output the sample size and
  # thresholds
  d <- tout_design(rho_0 = 0.5, rho_1 = 0.7, alpha_nom = 0.05, beta_nom = 0.1,
                   gamma_nom = 1, eta_0 = eta)
  return(c(d$n, d$thresholds))
}

# Apply our function to our etas
rs <- t(sapply(df$eta, get_results))
```

```
df$n <- rs[,1]; df$dif <- rs[,3] - rs[,2]

# Plot the results
ggplot(df, aes(eta)) + geom_line(aes(y=n)) +
  geom_line(aes(y = dif), linetype=2) +
  xlab(expression(paste("Probability of incorrect decision following a
                          pause outcome, ", eta))) +
  ylab("Sample size") +
  theme_minimal()
```

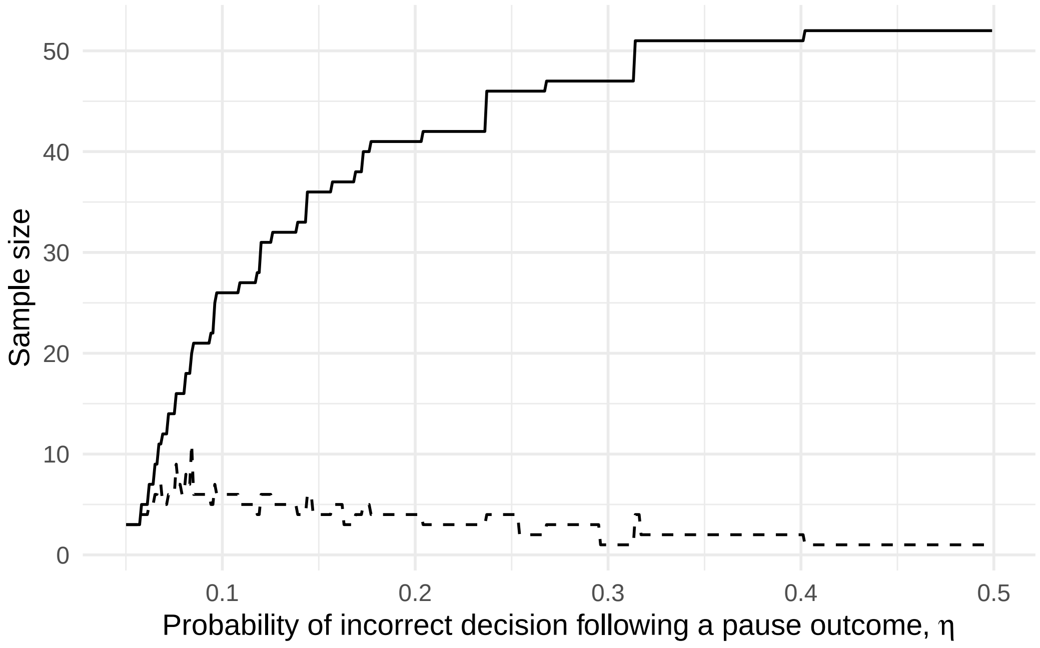


```
#ggsave("../paper/figures/eta_ns.pdf", height=9, width=14, units="cm")
```

From this we see the general trend, and can note that for a significant reduction in sample size we need to assume a very strong ability to make the correct progression decisions following a *pause* outcome. To examine sensitivity to an incorrect assumption of η, we take each of these optimal designs and find their error rates when $\eta = 0.5$

```
get_true_ocs <- function(z) {
  # z = vector (samples size, lower threshold, upper threshold)
  # Return the operating characteristics for any given design z when eta = 0.5
  d <- tout_design(rho_0 = 0.5, rho_1 = 0.7, alpha_nom = 0.05, beta_nom = 0.1,
```

```
                   gamma_nom = 1, eta_0 = 0.5, n = z[1], x = c(z[2], z[3]))
  c(d$alpha, d$beta)
}

# Apply the function to the designs generated in the previous analysis
ocs <- t(apply(rs, 1, get_true_ocs))
df$a <- ocs[,1]; df$b <- ocs[,2]

# Plot the results
ggplot(df, aes(eta)) +
  geom_line(aes(y = a, linetype = "b")) +
  geom_line(aes(y = b, linetype = "c")) +
  xlab(expression(paste("Assumed probability of incorrect decision following
                        a pause outcome, ", eta))) +
  ylab("Error rate") +
  scale_linetype_manual(name = "",
                       values = 1:2,
                       labels =c(expression(alpha), expression(beta))) +
  theme_minimal()
```

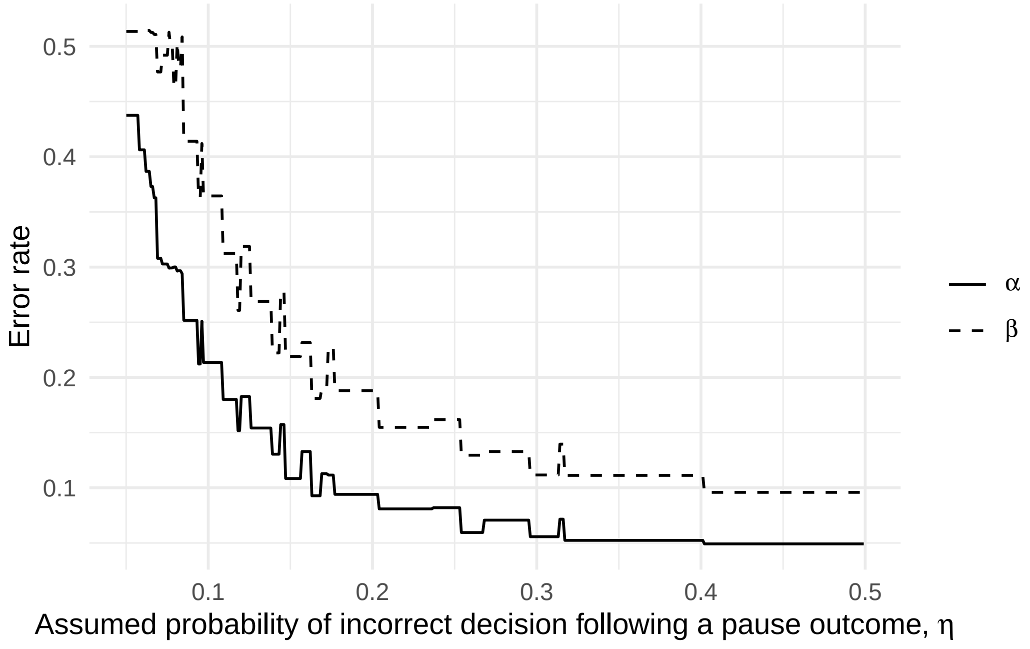


```
#ggsave("../paper/figures/eta_true_ocs.pdf", height=9, width=14, units="cm")
```

For example, compare the sample size saving and error rate inflation resulting from assuming $\eta = 0.2$ when in fact $\eta = 0.5$:

```
df[df$eta %in% c(0.4991, 0.2001),]
```

```
       eta  n dif          a         b
151 0.2001 41   4 0.09410551 0.1879656
450 0.4991 52   1 0.04918532 0.0959314
```

Incorporating other information

Assuming $\eta = 0.5$, we can help ensure *pause* outcome will occur with some desired probability when ρ is at the midpoint of the two hypotheses by constraining the OC γ. For example,

```
tout_design(rho_0 = 0.5, rho_1 = 0.7, alpha_nom = 0.05, beta_nom = 0.1,
            gamma_nom = 0.1)
```

```
Three-outcome design

Sample size: 187
Decision thresholds: 102 125

alpha = 0.04698389
beta = 0.09683741
gamma = 0.09704825

Hypotheses: 0.5 (null), 0.7 (alternative)
Modification effect range: 0 0
Error probability following an intermediate result: 0.5 0.5
```

If we want only a 10% chance of direct *go* or *stop* decisions when $\rho = (\rho_0 + \rho_1)/2 = 0.6$, we find we need to increase the sample size from $n = 52$ up to $n = 187$. Looking over a range of γ constraints gives:

```
df <- data.frame(gamma = seq(0.1, 1, 0.001))

get_results_gamma <- function(z) {
  d <- tout_design(rho_0 = 0.5, rho_1 = 0.7, alpha_nom = 0.05, beta_nom = 0.1,
                   gamma_nom = z)
  return(c(d$n, d$thresholds))
```

```
}

rs <- t(sapply(df$gamma, get_results_gamma))
df$n <- rs[,1]; df$dif <- rs[,3] - rs[,2]

ggplot(df, aes(gamma)) + geom_line(aes(y=n)) +
  geom_line(aes(y = dif), linetype=2) +
  xlab(expression(paste("Maximum probability of incorrect conclusive
                         decision, ", gamma, "*"))) +
  ylab("Sample size") +
  theme_minimal()
```

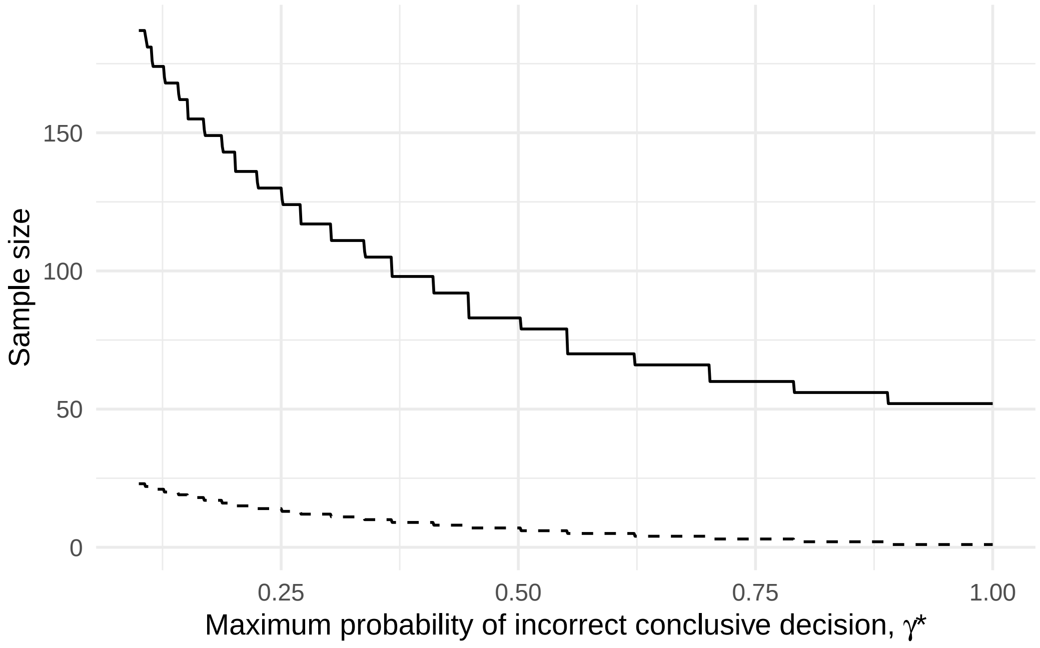


```
#ggsave("../paper/figures/gamma_ns.pdf", height=9, width=14, units="cm")
#ggsave("../paper/figures/gamma_ns.eps", height=9, width=14, units="cm", device = cairo_ps
```

For example, to move from $\gamma = 1$ to $\gamma = 0.4$ we need an increase in sample size of:

```
df[df$gamma %in% c(1, 0.4),]
```

```
    gamma  n dif
301   0.4 98   9
```

```
901    1.0 52    1
```

Allowing for adjustments

Finally, we consider how a three-outcome design works when we're planning on making adjustments. First, consider the case where the adjustment effect is known, i.e. when $\tau_{min} = \tau_{max} = \tau$.

```
# Look at a range of known adjustment effects
df <- data.frame(tau = seq(0, 0.125, 0.0005))

# For each tau, find the optimal design's sample size
ns <- sapply(df$tau, function(z) tout_design(rho_0 = 0.5, rho_1 = 0.7,
                                              alpha_nom = 0.05, beta_nom = 0.1,
                                              tau = c(z, z), max_n = 500)$n)

df$n <- ns

# Plot the results
ggplot(df, aes(tau)) + geom_line(aes(y=n)) +
  xlab(expression(paste("Known effect of adjustment, ", tau))) +
  ylab("Sample size") +
  theme_minimal()
```

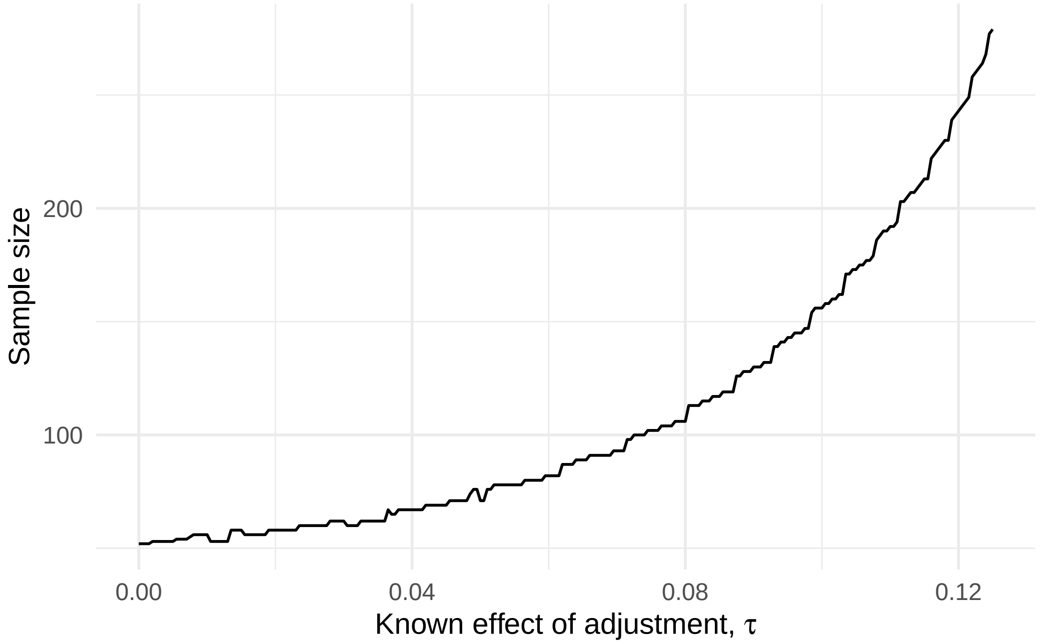


```
#ggsave("../paper/figures/tau_ns.pdf", height=9, width=14, units="cm")
```

We see that the larger the known adjustment effect, the more we need to inflate the sample size. To see the effect on sample size of allowing for a rage for the adjutsment effect τ, we find the optimal n for a range of lower bounds τ_{min} and interval widths $\tau_{max} - \tau_{min}$:

```
# Create a grid on adjustment effect interval lower bounds (l) and widths (w)
df <- expand.grid(l = seq(0, 0.1, 0.005),
                  w = seq(0, 0.05, 0.005))

ns <- apply(df, 1, function(z) tout_design(rho_0 = 0.5, rho_1 = 0.7,
                                            alpha_nom = 0.05, beta_nom = 0.1,
                                            tau = c(z[1], z[1] + z[2]),
                                            max_n = 700)$n)
df$n <- ns

ggplot(df, aes(l, w)) + geom_tile(aes(fill = n)) +
  scale_fill_continuous(type = "viridis") +
  xlab(expression(paste("Lower bound of adjustment effect, ", tau[min]))) +
  ylab(expression(paste("Width of adjustment effect interval, ",
                        tau[max] - tau[min]))) +
  theme_minimal()
```

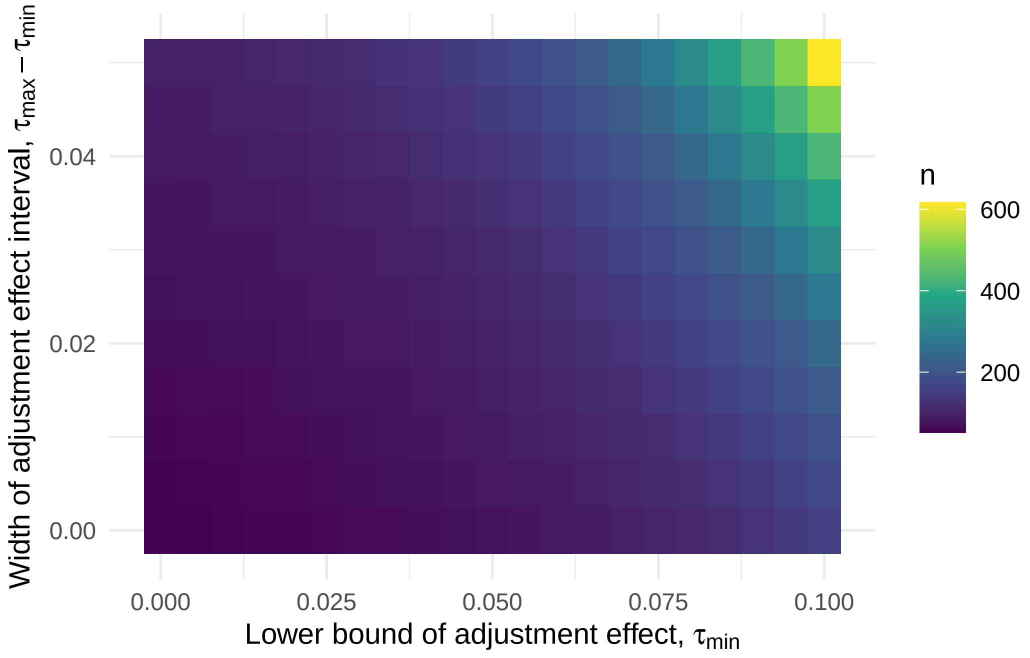


```
#ggsave("../paper/figures/tau_part_ns.pdf", height=9, width=14, units="cm")
```

We find that increasing τ_{max} while fixing τ_{min} further increases the sample size required. At the extremes, we find:

```
df[df$l == 0 & df$w == 0,]
```

```
  l w  n
1 0 0 52
```

```
df[df$l == 0 & df$w == 0.05,]
```

```
    l    w  n
211 0 0.05 93
```

```
df[df$l == 0.1 & df$w == 0,]
```

```
     l w   n
21 0.1 0 156
```

```
df[df$l == 0.1 & df$w == 0.05,]
```

```
      l    w   n
231 0.1 0.05 616
```

The figure suggests that sample size is being driven primarily by τ_{max}, but there is still some substantial variation due to τ_{min}. For example:

```
# Change lower bound from 0.1 to 0 while upper bound remains 0.15
tout_design(rho_0 = 0.5, rho_1 = 0.7, alpha_nom = 0.05, beta_nom = 0.1,
            tau = c(0.1, 0.15), max_n = 8000)$n
```

```
[1] 616
```

```
tout_design(rho_0 = 0.5, rho_1 = 0.7, alpha_nom = 0.05, beta_nom = 0.1,
            tau = c(0.0, 0.15), max_n = 8000)$n
```

```
[1] 851
```

```
# Change lower bound from 0.05 to 0.01 while upper bound remains 0.05
tout_design(rho_0 = 0.5, rho_1 = 0.7, alpha_nom = 0.05, beta_nom = 0.1,
            tau = c(0.05, 0.05), max_n = 8000)$n
```

```
[1] 71
```

```
tout_design(rho_0 = 0.5, rho_1 = 0.7, alpha_nom = 0.05, beta_nom = 0.1,
            tau = c(0.01, 0.05), max_n = 8000)$n
```

```
[1] 87
```

Finally, we can examine the effect of η when allowing adjustments by varying η whilst fixing $\tau_{min} = 0.05$ and $\tau_{max} = 0.1$:

```
df <- data.frame(eta = seq(0.0501, 0.5, 0.001))

ns <- sapply(df$eta, function(z) tout_design(rho_0 = 0.5, rho_1 = 0.7,
```

```
                                                    alpha_nom = 0.05, beta_nom = 0.1,
                                                    eta_0 = z, tau = c(0.05, 0.1),
                                                    max_n = 500)$n)
df$n <- ns

ggplot(df, aes(eta)) + geom_line(aes(y=n)) +
  xlab(expression(paste("Probability of incorrect decision following
                           a pause outcome, ", eta))) +
  ylab("Sample size") +
  theme_minimal()
```

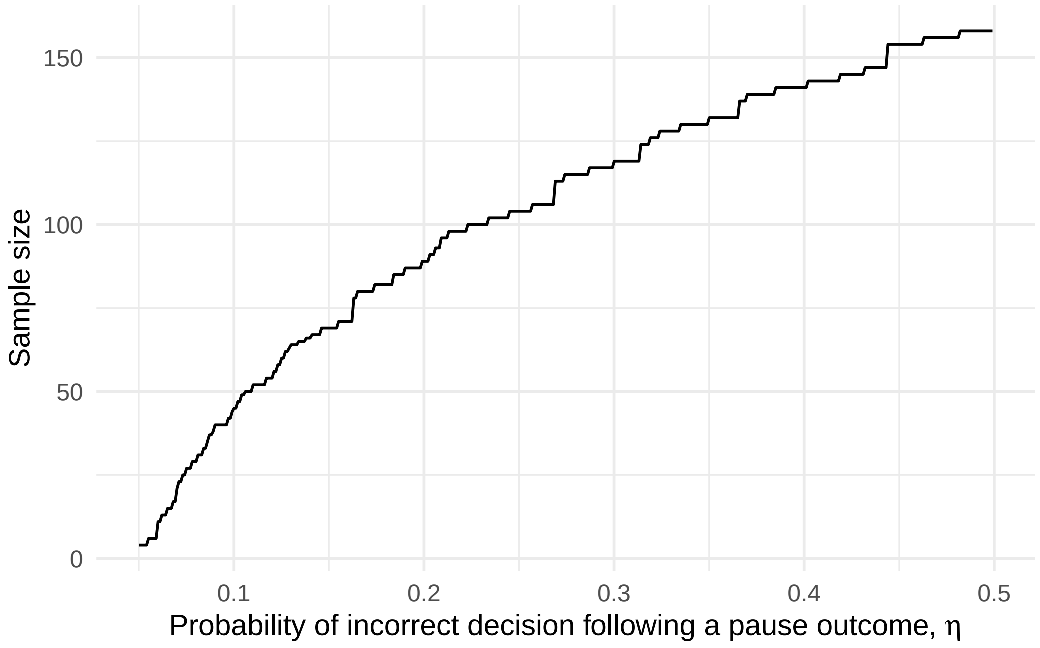

We see that a lower η can give more reasonable sample sizes. Unlike in the non-adjustment case, we might argue $\eta < 0.5$ is plausible because even if we can't learn anything about ρ beyond its sufficient statistic $\hat{\rho}$, we might have learnt about τ from the pilot (i.e. we have a good idea of what adjustment we can make and what effect it will have). But this can only take us so far, since we need to know about the adjusted parameter $\rho + \tau$ so there will always be some residual uncertainty. Thus, extreme cases like the one above where η is so low that the optimal design becomes $n = 2$ with a almost guaranteed pause result (following which we almost always make the right decision) aren't realistic.

Relaxing error rate constraints while fixing n

We have focused on how the required sample size changes with the various parameters in our problem while keeping error rates fixed. Here, we instead fix the sample size and look at changes in error rates. For example, suppose we fix $n = 30$ and $\eta = 0.5$. We calculate the error rates for every possible choice of progression criteria x_0, x_1:

```
df <- expand.grid(x_0 = 0:30,
                  x_1 = 0:30)

df <- df[df$x_1 >= df$x_0,]

df <- cbind(n=30, df)

get_true_ocs <- function(z) {
  # z = vector (samples size, lower threshold, upper threshold)
  # Return the operating characteristics for any given design z when eta = 0.5
  d <- tout_design(rho_0 = 0.5, rho_1 = 0.7, alpha_nom = 0.05, beta_nom = 0.1,
                   gamma_nom = 1, eta_0 = 0.5, n = z[1], x = c(z[2], z[3]))
  c(d$alpha, d$beta, d$gamma)
}

ocs <- t(apply(df, 1, get_true_ocs))
df <- cbind(df, ocs)
names(df)[4:6] <- c("alpha", "beta", "gamma")

ggplot(df, aes(alpha, beta)) + geom_point(aes(colour = gamma)) +
  geom_point(data = df[df$x_0 == 19 & df$x_1 == 19,], shape = 2,
             size = 5, colour = "blue") +
  geom_point(data = df[df$x_0 == 17 & df$x_1 == 17,], shape = 2,
             size = 5, colour = "green") +
  geom_point(data = df[df$x_0 == 15 & df$x_1 == 20,], shape = 2,
             size = 5, colour = "red") +
  scale_colour_continuous(type = "viridis", name = expression(gamma)) +
  xlab(expression(alpha)) + ylab(expression(beta)) +
  theme_minimal()
```

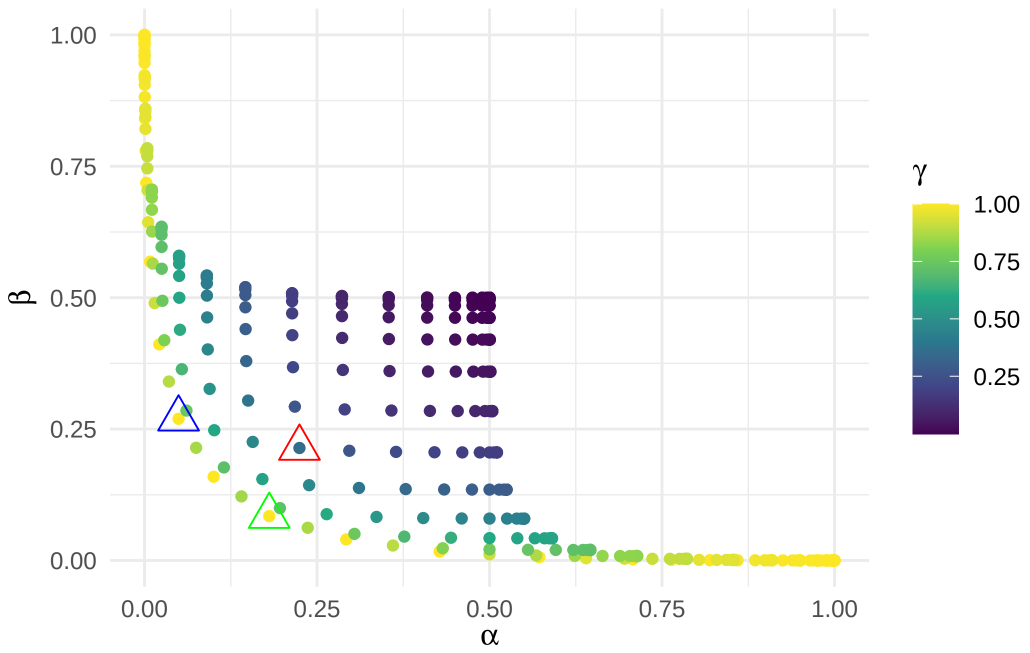

This again shows that moving from a two-outcome to three-outcome design does not improve efficiency, but rather that it necessitates a reduction in α and/or β in order to reduce γ. For example, $x_0 = x_1 = 19$ gives error rates of $\alpha = 0.05, \beta = 0.27, \gamma = 1$. The most efficient way to reduce β is to keep the two-outcome design but change the threshold, e.g. to $x_0 = x_1 = 17$ which gives $\alpha = 0.18, \beta = 0.08, \gamma = 1$. Moving to a three-outcome design should only be done to reduce γ; for example, thresholds of $x_0 = 15, x_1 = 20$ will give $\alpha = 0.22, \beta = 0.21, \gamma = 0.35$.

Illustrative figures

Sargent sampling distribution illustration:

```
ggplot(data.frame(x = c(-10, 10)), aes(x)) +
  # Null
  stat_function(fun = dnorm, geom = "line", aes(linetype="a")) +
  stat_function(fun = dnorm, geom = "area", fill = "orange", alpha = 0.4,
                xlim = c(2, 7)) +
  stat_function(fun = dnorm, geom = "area", fill = "red", alpha = 0.4,
                xlim = c(1, 2)) +

  # Alternative
  stat_function(fun = dnorm, args = list(mean = 2.5), geom = "line",
```

```
               aes(linetype="b")) +
stat_function(fun = dnorm, args = list(mean = 2.5),
              geom = "area", fill = "darkgreen", alpha = 0.4,
              xlim = c(-3, 1)) +
stat_function(fun = dnorm, args = list(mean = 2.5),
              geom = "area", fill = "blue", alpha = 0.4, xlim = c(1, 2)) +

geom_label(aes(x = 0.6, y = 0.22, label = "lambda"), parse = T,
           fill = "red", alpha = 0.4) +
geom_label(aes(x = 2.4, y = 0.17, label = "delta"), parse = T,
           fill = "blue", alpha = 0.4) +
geom_label(aes(x = 0.2, y = 0.1, label = "beta[a]"), parse = T,
           fill = "darkgreen", alpha = 0.4) +
geom_label(aes(x = 2.6, y = 0.06, label = "alpha[a]"), parse = T,
           fill = "orange", alpha = 0.4) +

geom_text(aes(x=1, y=-0.04, label = "x[0]"), parse = T) +
geom_text(aes(x=2, y=-0.04, label = "x[1]"), parse = T) +

scale_linetype_manual(values = c(1,2),
                      labels = c(expression(rho[0]),
                                 expression(rho[1])), name="") +

xlim(-4, 6.5) + theme_void() + theme(legend.position="bottom")
```

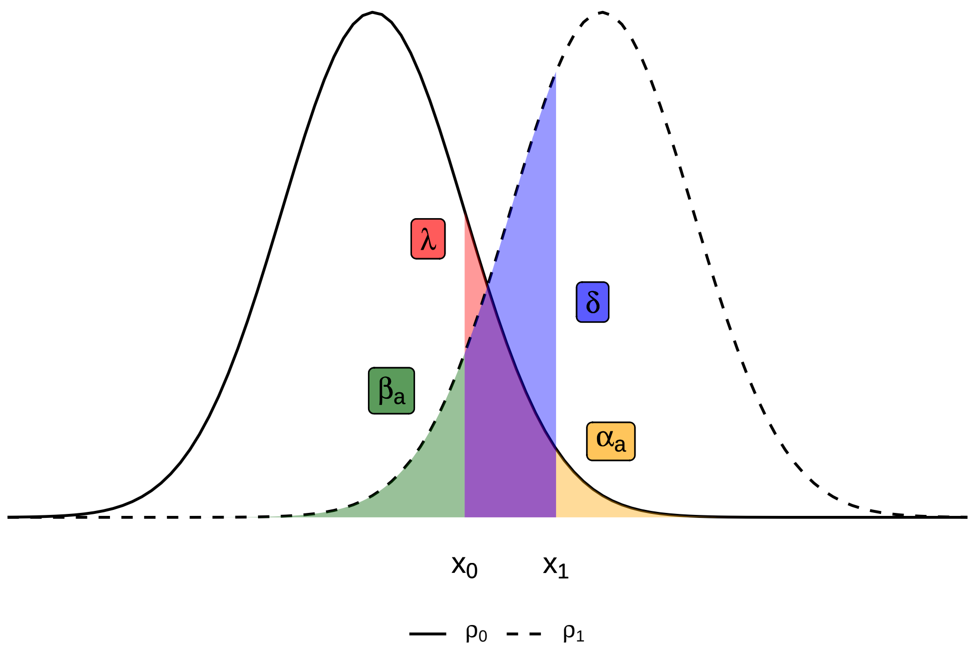


```
#ggsave("../paper/figures/Sarg_ocs.pdf", height=4.5, width=14, units="cm")
```

Storer sampling distribution illustration:

```
p1 <- ggplot(data.frame(x = c(-10, 10)), aes(x)) +
  # Null
  stat_function(fun = dnorm, geom = "line", aes(linetype = "a")) +
  stat_function(fun = dnorm, geom = "area", fill = "orange", alpha = 0.4,
                xlim = c(1, 7)) +

  # Alternative
  stat_function(fun = dnorm, args = list(mean = 2.5), geom = "line",
                aes(linetype = "b")) +
  stat_function(fun = dnorm, args = list(mean = 2.5),
                geom = "area", fill = "darkgreen", alpha = 0.4,
                xlim = c(-3, 2)) +

  geom_label(aes(x = 0.2, y = 0.12, label = "beta[b]"), parse = T,
             fill = "darkgreen", alpha = 0.4) +
  geom_label(aes(x = 2.6, y = 0.08, label = "alpha[b]"), parse = T,
             fill = "orange", alpha = 0.4) +
```

```
  geom_text(aes(x=1, y=-0.04, label = "x[0]"), parse = T) +
  geom_text(aes(x=2, y=-0.04, label = "x[1]"), parse = T) +

  stat_function(fun = dnorm, geom = "line", aes(linetype = "c"),
                xlim = c(0,0)) +

  scale_linetype_manual(values = c(1, 2, 3),
                        labels = c(expression(rho[0]), expression(rho[1]),
                                   expression(rho[m])), name="") +

  xlim(-4, 6.5) + theme_void() + theme(legend.position="bottom")

p1
```

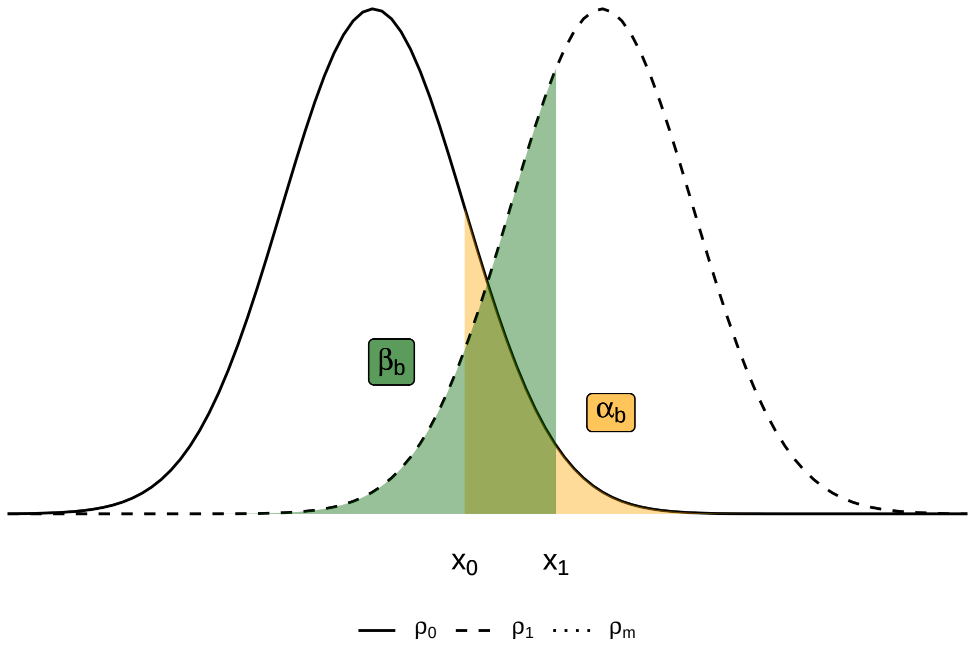


```
p2 <- ggplot(data.frame(x = c(-10, 10)), aes(x)) +
  # Midpoint
  stat_function(fun = dnorm, args = list(mean = 1.25), geom = "line",
                linetype=3) +
  stat_function(fun = dnorm, args = list(mean = 1.25),
                geom = "area", fill = "red", alpha = 0.4, xlim = c(-3, 1)) +
  stat_function(fun = dnorm, args = list(mean = 1.25),
```

```
                  geom = "area", fill = "blue", alpha = 0.4, xlim = c(2, 7)) +

  geom_label(aes(x = -0.9, y = 0.12, label = "gamma[L]"), parse = T,
             fill = "red", alpha = 0.4) +
  geom_label(aes(x = 3.4, y = 0.12, label = "gamma[U]"), parse = T,
             fill = "blue", alpha = 0.4) +
  xlim(-4, 6.5) + theme_void()

p2
```

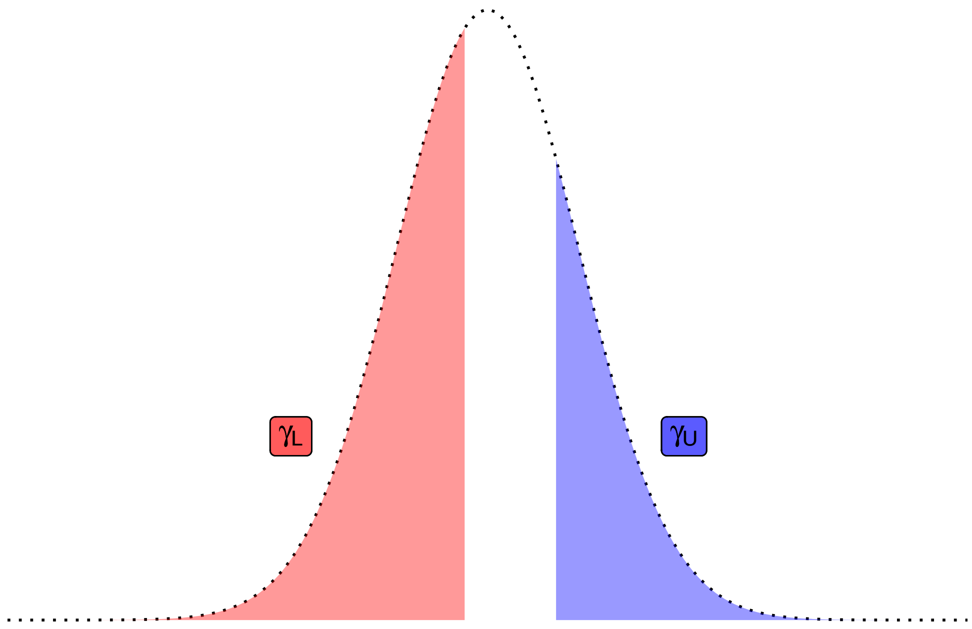


```
#library(patchwork)
#p2/p1

#ggsave("../paper/figures/Stor_ocs.pdf", height=8, width=14, units="cm")
```
